# High DNA stability in white blood cells and buffy coat lysates stored at ambient temperature under anoxic and anhydrous atmosphere

**DOI:** 10.1371/journal.pone.0188547

**Published:** 2017-11-30

**Authors:** Anne-Lise Fabre, Aurélie Luis, Marthe Colotte, Sophie Tuffet, Jacques Bonnet

**Affiliations:** 1 Imagene, R&D department, Université de Bordeaux, ENSTBB, 146 Rue Léo Saignat, Bordeaux, France; 2 Imagene, production platform, Rue Henri Desbruères, Genopole campus 1, Bât 6, Evry, France; 3 Institut Bergonié- Université de Bordeaux, INSERM U1218, 229 Cours de l'Argonne, Bordeaux, France; Charles University, CZECH REPUBLIC

## Abstract

Conventional storage of blood-derived fractions relies on cold. However, lately, ambient temperature preservation has been evaluated by several independent institutions that see economic and logistic advantages in getting rid of the cold chain. Here we validated a novel procedure for ambient temperature preservation of DNA in white blood cell and buffy coat lysates based on the confinement of the desiccated biospecimens under anoxic and anhydrous atmosphere in original hermetic minicapsules. For this validation we stored encapsulated samples either at ambient temperature or at several elevated temperatures to accelerate aging. We found that DNA extracted from stored samples was of good quality with a yield of extraction as expected. Degradation rates were estimated from the average fragment size of denatured DNA run on agarose gels and from qPCR reactions. At ambient temperature, these rates were too low to be measured but the degradation rate dependence on temperature followed Arrhenius’ law, making it possible to extrapolate degradation rates at 25°C. According to these values, the DNA stored in the encapsulated blood products would remain larger than 20 kb after one century at ambient temperature. At last, qPCR experiments demonstrated the compatibility of extracted DNA with routine DNA downstream analyses. Altogether, these results showed that this novel storage method provides an adequate environment for ambient temperature long term storage of high molecular weight DNA in dehydrated lysates of white blood cells and buffy coats.

## Introduction

Blood samples (or liquid biopsies) are seen as key biospecimens to drive the future of personalized medicine since they can be used to detect, diagnose and/or monitor diseases through genomic and transcriptomic analyses. They may be processed to produce serum, plasma, white blood cells (WBC) or buffy coat (BC) fractions. In particular, human WBC and BC fractions provide concentrated sources of nucleated cells (WBC, in buffy coat, are approximately 10 times more concentrated than in whole blood) from which to extract DNA [1,2]. Therefore, they are easier to store and ship than whole blood.

WBC and BC fractions are usually stored in the cold [3]. However, the exponentially growing number of samples to preserve leads to problems of costs (energy, maintenance, space or investments in freezers) and risk management. In addition, maintaining cold chain to avoid degradation during shipment is expensive. At last, several recent studies have shown problems in DNA extraction yield from stored frozen blood samples over time [4,5,6], strong modification of DNA methylation [7] and risks of loss rise with freeze-thawing [8,9].

In a context where biobanks and biological resources centers are looking for operational financial and social, sustainability, these drawbacks make appealing ambient temperature storage and shipping.

Ambient temperature storage of blood-derived biospecimens for DNA analysis, however, requires that DNA stability and compatibility with downstream analyses are preserved. In solution or in dead tissues, the main non-enzymatic DNA degradation factor is water (for reviews about DNA degradation see for instance [10,11,12,13]). Water either acts directly or catalyzes mechanisms such as depurination which is generally the fastest DNA degradation process. Depurination is rapidly followed by chain breaking at the depurination site [14]. Water is also necessary to the generation of reactive oxygen species (ROS) which DNA is particularly vulnerable to. In particular, in solution, in the presence of oxygen and traces of metal ions such as Cu^2+^ or Fe^3+^, the rate of formation of 8-oxoguanine (guanine being the most easily oxidized DNA element) in purified DNA can be of the same order of magnitude as that of depurination [15,16]. Guanine oxidation, however does not accelerate depurination and so, does not promote chain breaking [17,18]. Another oxidation process can be brought by atmospheric ozone [19,20]. As a consequence, dehydration has been the basis of several procedures for ambient temperature storage of purified DNA (for instance, freeze-drying, vacuum drying, use of soluble or solid matrices, reviewed in[21]).

When dried, DNA is fairly stable [10,22], as exemplified by the fact that commercial DNA is commonly shipped freeze-dried at ambient temperature, but remains sensitive to atmosphere exposure [23,24,25]. The same is true for DNA in freeze-dried tissues[22,26], blood or cells deposited on solid adsorbing matrixes such as FTA paper [27], Guthrie cards [28], or ViveST™ (ViveBio, Alpharetta, GA) absorbing matrix.

There are commercial products for ambient temperature storage of blood and blood-derived fractions in liquid form (HEMAgene® for BC DNA, PAXgene and Tempus™ for blood DNA or RNA, AquaGenomic frozen blood DNA, RNAgard® blood, RNA*later*®, DNA/RNA Shield™ Blood collection, Streck Cyto-Chex™ BCT Storage and Transport Reagent…) but they have not been fully validated for long term storage at ambient temperature. In addition, because large volumes of stabilization solutions are necessary, their use require an increase of space (e.g between 2.5 and 9 volumes of blood depending on the product).

In this context, an innovative procedure, where desiccated purified DNA or RNA, are maintained under an anoxic and anhydrous atmosphere inside hermetic minicapsules has been developed by Imagene [29,30,31] and independently validated [32,33]. This process allows a standalone storage (no need for humidity or temperature controls) and enables long term stability of DNA at ambient temperature (over centuries).

Here we present data extending Imagene's preservation process to buffy coat and white blood cells lysates for genomic DNA downstream analysis. We estimated the quantity and the quality of DNA extracted from these biospecimens over time under both real time storage conditions and through accelerated ageing. We used the Arrhenius’ model to estimate ambient temperature DNA stability in the biospecimens.

## Materials and methods

### Ethics statement

The data regarding DNA stability of WBC and BC lysates presented in this study relate to projects that have been formally approved by the “Comité de protection des personnes Sud Ouest et Outre Mer III”, including use of blood and blood-derived samples for this study.

"Etablissement français du sang" (EFS, France) is a French national establishment that is authorized to collect blood samples from adult volunteer donors for both therapeutic and non-therapeutic uses. The donations were collected in accordance with the French blood donation regulations and ethics and with the French Public Health Code (art L1221-1). Blood samples were anonymized according to the French Blood Center (EFS) procedure. Volunteer donors signed written informed consents before blood collection. EFS authorized Imagene to perform this study and provided de-identified blood and blood-derived samples for non-therapeutic use.

### Blood fractions sources and preparation

#### White blood cells

Human whole blood samples were collected in EDTA anti-coagulation tubes of 5 mL per donor and tested for biosafety at the "Etablissement français du sang" (EFS, France). After validation, blood samples were shipped to the laboratory at ambient temperature within 24 hours and preserved at– 80°C until white blood cells preparation.

Two tubes of 5 mL blood were thawed and pooled. They received 30 mL of red blood cell lysis solution (10 mM Tris-HCl pH 7.6, 10 mM NaCl, 5 mM MgCl_2_) at ambient temperature. The solution was homogenized by 20 inversions. After 15 min incubation at ambient temperature, the tubes were centrifuged at 2000 g for 5 min at ambient temperature to pellet the WBC and the supernatant was discarded. A second red blood cell lysis step was performed on the WBC pellet following the same protocol on the WBC pellet. The latter was preserved at -80°C until use.

#### Buffy coats

Buffy coats were prepared and tested for biosafety by EFS, shipped to the laboratory at ambient temperature within 24 hours, aliquoted, and kept at -80°C until use. According to EFS, each buffy coat is prepared by centrifugation from one donor and contains concentrated platelets (95% of platelets in the blood sample) and almost all white blood cells of the sample. They are stored in phosphate citrate and dextrose. They appeared in red color suggesting a strong contamination in red blood cells.

#### Buffy coats and white blood cells lysates encapsulation

**Fig 1:**
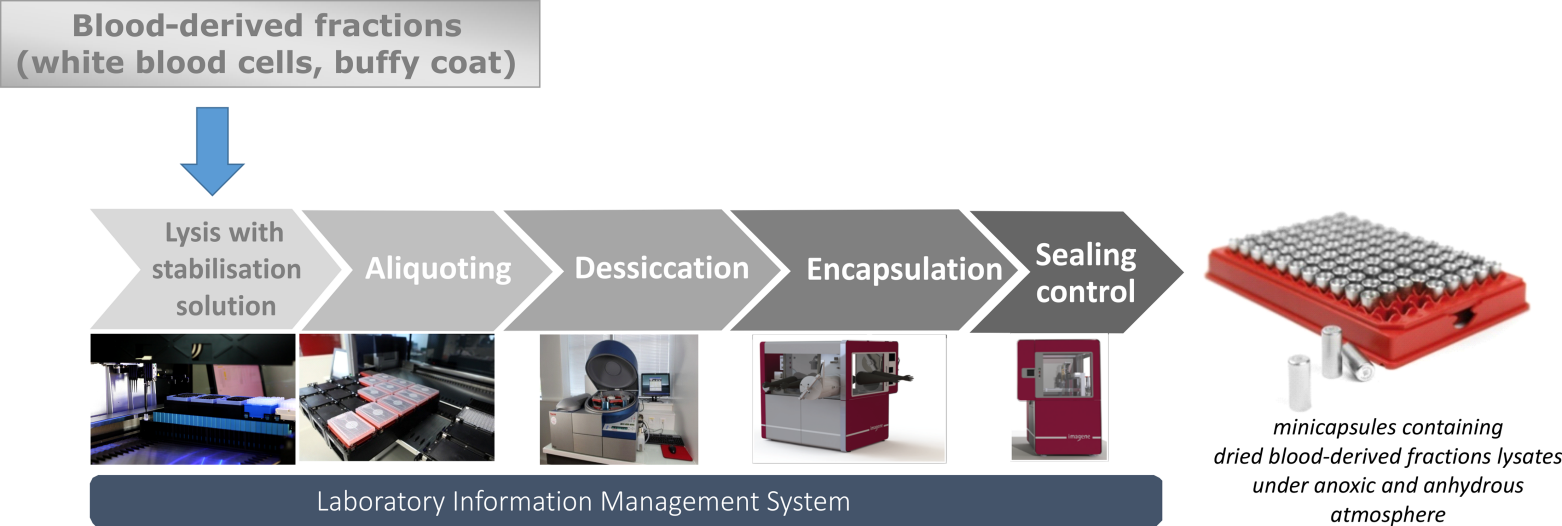
Imagene process for encapsulation of blood-derived fractions (white blood cells and buffy coat). The samples are lysed with proprietary solutions, aliquoted in minicapsules, then desiccated under vacuum. After incubation and sealing under anhydrous and anoxic atmosphere, each minicapsules is checked for leakage by mass spectrometry and then stored in a SBS-format plate at ambient temperature.

WBC and BC samples were thawed and lysed in either of 2 different Imagene proprietary solutions. Imagene proprietary solutions are part of the Imagene process and are necessary, as much as the encapsulation process, to replicate the study. The lysates were homogenized by inversions. Either 92 μL of WBC lysate or 100 μL of BC lysate were aliquoted in glass insert fitted in open minicapsules. The samples were dried under vacuum and left in a glove box under an inert anhydrous argon/helium atmosphere. Then, a cap was added to the minicapsules and closed by laser welding. Finally, each minicapsules was checked for leakage by mass spectrometry (Fig 1) [31].

Several batches were produced for this study. We define a batch of minicapsules as a set of capsules coming from the same source (same pool of donor), treated according to the same process (lysis and encapsulation) and at the same time. For each batch, 10 minicapsules of WBC and 11 minicapsules of BC were produced from 1 mL of whole blood. Based on an average number of 5 million WBC per mL of whole blood (assumption based on the cell number per mL of blood: about 8.7 million of nucleated cells/mL [34]), we considered that each aliquot contained about 5.2 μg of genomic DNA.

### Real time and accelerated degradation studies

For each kinetic, all the capsules were taken from the same batch. For real time kinetics, WBC minicapsules and BC minicapsules were stored at ambient temperature (22°C ± 4°C) either in the native minicapsules format or opened to air. For this later condition, minicapsules were left at ambient temperature so that dried blood-fraction were exposed to the atmosphere of the laboratory during the kinetic (~ 50% relative humidity). At each time point, 3 minicapsules were collected and stored at -20°C until DNA extraction and analysis or used extemporaneously.

For accelerated DNA degradation, minicapsules were heated at temperatures ranging from 50°C to 130°C. One kinetic was conducted at 70°C under air at 50% relative humidity in order to mimic atmospheric conditions at this temperature. For this kinetic, open and intact minicapsules were put in microtubes placed in a bottle with a saturated BrNa solution that maintained a 50% relative humidity. At each time point of the kinetics, 3 minicapsules were retrieved, quickly cooled on ice and then stored at -20°C until DNA extraction and analysis or used extemporaneously. The "t_0_" time corresponds to a 2–5 min incubation required for the minicapsules to reach the nominal temperature of the kinetics.

### DNA extraction

For each time point of the kinetics, the minicapsules were removed from the heating block or from -20°C freezer, left at ambient temperature for 5 min, then opened with a “shellOpener” and rehydrated as described below.

For WBC, 100 μL of nuclease-free water were added to the dried lysates. After a 15 min incubation at 37°C, the 100 μL of resuspended lysate were aliquoted in a 2 ml plastic tube. Thirty-three μL of the protein precipitation solution without using proteinase K were added to the lysate and the DNA was extracted following the Puregene protocol (Gentra, Qiagen, Hilden Germany). The DNA was resuspended in 30 μL 1× TE (10 mM Tris-HCl, 1 mM EDTA, pH 8) and stored at 4°C before analysis.

For BC, 100 μL of 1× TE were added to the dried lysates. After a 45 min incubation at 37°C, the DNA was extracted following the Puregene protocol (Gentra, Qiagen, Hilden Germany) starting with the addition of 250 μL of cell lysis solution and 30 μg of proteinase K followed by a 30 min incubation at 50°C. The DNA was resuspended in 30 μL of the DNA hydration solution and stored at 4°C before analysis.

### DNA yield and purity

We measured the concentration and absorbance ratios (A260 nm / A280 nm and A260 nm / A230 nm) of the extracted DNA samples by UV spectrophotometry (Nanodrop, ThermoFisher Scientific, Waltham, MA) to evaluate the extraction yield and the purity of the DNA.

### DNA sizing on agarose gels

DNA size profiles were visualized on a non-denaturing agarose gel electrophoresis (AGE) after heat denaturation (95°C for 5 min) to evidence single strand breaks. The gels were stained for one hour with ethidium bromide (500 ng/μL) and photographed with a digital imaging device under UV light (Quantum ST4 1000, Vilber Lourmat, Collégien, France). The images were analyzed with the Bio1D image analysis software (Vilber Lourmat, Collégien, France).

At each time point of storage, t, at a given temperature, T, we determined the length of the molecule corresponding to the maximum of the mass distribution of the DNA population (i.e. to the maximum staining intensity of the gel), L_max_ [23].

It is known, that heat-denatured mammalian DNA brought back at ambient temperature contains a small but non negligible proportion of double strands because of internal foldbacks [35]. Moreover the sizes were measured by reference to non-denatured molecular weight markers, so, hereafter we refer to these sizes as "apparent" sizes.

### Estimation of degradation rate from electrophoresis data

Degradation rate constants estimated through AGE refer to chain breaking events. A degradation rate for a given temperature (T), k_T_, was determined by using the model of random degradation previously validated [23].

This model states that:

$$L_{max}∼\frac{1}{number\;of\;cuts\;per\;nucleotides}$$

or

$$L_{max}∼\frac{1}{P_{cut}}$$

where P_cut_ is the probability of a breaking event per nucleotide during a random degradation process i.e. the number of cuts per nucleotide.

If N_0_ is the initial number of cuts/nucleotide at t_0_ and N_t_ is the number of cuts added at time t, then we have:

$$L_{max}=\frac{1}{\left(N_{0}+N_{t}\right)}$$

or

$$\frac{1}{L_{max}}=N_{0}+N_{t}=N_{0}+k_{T}.t$$ (1)

N_0_ being constant, 1/ L_max_ is a linear function of t, described by a straight line with a slope equal to k_T_. Therefore, k_T_ was determined for each temperature and we plotted the -log k_T_ values as a function of 1/T according to the Arrhenius equation:

$$k_{T}=A.e^{\left(-\frac{E_{a}}{RT}\right)}$$ (2)

where:

- R = 8.31 J·mol^-1^·K^-1^,
- A is the pre-exponential factor and
- Ea is the activation energy of the degradation reaction.

We evaluated the apparent Ea and the apparent degradation rate at ambient temperature by extrapolation from the equation of the obtained Arrhenius straight line.

### Quantitative PCR experiments

For BC, we used the same batch of capsules for both quantitative PCR (qPCR) and AGE analyses. For WBC, we used two different batches in order to have a sufficient number of capsules.

One minicapsule was used for each time point leading to a total random-use of 12 BC and 12 WBC minicapsules. Two microliters of extracted DNA from WBC and BC minicapsules, corresponding to an average of 169 ± 59 ng and 206 ± 103 ng of WBC and BC DNA respectively, were aliquoted as templates for the qPCR reactions. They were used undiluted or 10 fold and 100 fold diluted. The qPCR reactions were run on the CFX96 (Bio-Rad, Hercules, CA, USA) using the “qPCR Fast Start Essential DNA green master kit” with a SYBRgreen-based detection (Roche, Bâle, Switzerland). The DNA amounts were not normalized before sampling for qPCR (see below”Estimation of degradation rate from qPCR data”).

Two amplicons of 1064 bp and a 93 bp of the *TAF1L* gene (TATA-box binding protein associated factor 1 like, Gene ID: 138474) were targeted. PCR cycles were as follow: 10 min at 90°C then 40 cycles of (15 s at 95°C; 15 s at 60°C; 60 s at 72°C). The primers sequences were

For-5' agactcggacagcgaggaa / Rev-5' cggagacacccagcatatca for the 1064 pb fragment and

For-5' tgcaggcacttgagaacaac / Rev-5' aaccctgtcttgtccgaatg for the 93 pb fragment. They were supplied by Eurobio, Les Ulis, France. The 3 ten-fold dilutions were used to estimate the PCR efficiency for each sample and each amplicon. In this case, the samples and their dilutions were analyzed independently and defined as “standards” of known quantities (see Results).

### Estimation of chain breaking rate from qPCR data

According to our previous work [23], it is possible to estimate the average fragment size L_max_ of a given DNA population if the chain breaking process is random, by quantifying the amplifiable copy numbers of 2 amplicons of different sizes.

The combination of the equations of a qPCR reaction and of a random degradation process, for the amplicon 1, gives:

$$N_{1,t}=N_{1,t0}.e^{-k_{T}.L_{1}.t}$$

$$N_{1,q}=N_{1,t}{\left(1+E\right)}^{C_{q}}$$

$$N_{1,q}=N_{1,t0}{.e}^{-k_{T}.L_{1}.t}{.(1+E)}^{C_{q}}$$

where:

- N_1,t0_, N_1,t_ and N_1,q_ are the copy numbers of the amplicon 1, at the beginning, at the time t of the degradation kinetic and at the threshold of the qPCR respectively,
- k_T_ is the DNA degradation rate constant at the temperature T,
- L_1_ is the size of the amplicon "1" and
- C_q_ is the quantification cycle, according to the RDML data standard [36].
- E is the PCR efficiency which can be considered, here, as constant and equal to 1 (see Results).

Therefore,

$$C_{1,q}.ln2=k_{T}.L_{1}.t+\ln\left(N_{1,q}\right)-\ln\left(N_{1,t0}\right)$$

and

$$C_{1,q}=\frac{k_{T}.L_{1}}{ln2}.t+\frac{1}{ln2}.ln(\frac{N_{1,q}}{N_{1,t0}})$$

The same can be written for amplicon 2. The difference member to member of both expressions gives:

$$\Delta C_{q}=C_{1,q}-C_{2,q}=\frac{k_{T}.(L_{1}-L_{2})}{ln2}t+\frac{1}{ln2}.\ln\left(\frac{N_{1,q}}{N_{1,t0}}\right)-\frac{1}{ln2}.ln(\frac{N_{2,q}}{N_{2,t0}})$$

$$\Delta C_{q}=\frac{k_{T}(L_{1}-L_{2})}{ln2}t+\frac{1}{ln2}\ln\left(\frac{N_{1,q}}{N_{1,t0}}\times \frac{N_{2,t0}}{N_{2,q}}\right)$$ (3)

Since N_1,t0_, N_2,t0_, N_1,q_ and N_2,q_ are constants, ΔCq values plotted as a function of time must be fitted by a straight line, with a slope equal to

$$\frac{k_{T}.(L_{1}-L_{2})}{ln2}$$

## Results

### DNA quantity and quality from dried blood-derived products in WBC minicapsules and BC minicapsules

We extracted and analyzed the DNA from more than 100 minicapsules. Each one of them was aliquoted with the same volume of blood-derived fractions (see “Blood fractions sources and preparation”). Tables 1 and 2 give the DNA extraction yields, the absorbance ratios A260 nm/ A280 nm and A260 nm/ A230 nm for DNA extracted from WBC and BC minicapsules respectively. The same data are displayed graphically in S1 Fig.

**Table 1:** Quantity and quality of DNA extracted from encapsulated white blood cell assessed by spectrophotometric measurements: Yields and absorbance ratios for DNA extracted from white blood cells.

Temperature	Storage time	Yield (μg)		A_260 nm_/ A_280 nm_		A_260 nm_/ A_230 nm_	
		Mean (SD)		Mean (SD)		Mean (SD)	
Ambient	months						
	0	6.60	(4.10)	1.85	(0.01)	2.21	(0.02)
	12	10.20	(2.60)	1.84	(0.00)	2.19	(0.02)
	15	9.10	(2.80)	1.93	(0.13)	2.63	(0.65)
70°C	hours						
	0	3.20	(1.89)	1.87	(0.03)	2.16	(0.13)
	12	3.86	(1.66)	1.84	(0.03)	2.09	(0.14)
	72	2.38	(0.50)	1.85	(0.02)	1.91	(0.09)
	96	2.25	(0.37)	1.87	(0.01)	2.03	(0.15)
	144	2.98	(0.97)	1.84	(0.04)	1.90	(0.16)
90°C	min						
	0	1.66	(0.05)	1.84	(0.02)	2.32	(0.02)
	24	1.32	(0.02)***	1.87	(0.01)	2.50	(0.13)
	96	1.25	(0.08)**	1.83	(0.04)	2.28	(0.39)
	168	1.10	(0.11)**	1.88	(0.02)	2.04	(0.11)
	264	1.24	(0.30)	1.84	(0.02)	2.23	(0.13)
	360	1.37	(0.33)	1.85	(0.00)	2.49	(0.34)
110°C	min						
	0	2.03	(0.16)	1.83	(0.03)	1.86	(0.13)
	60	1.85	(0.11)	1.86	(0.02)	2.12	(0.28)
	180	1.73	(0.09)*	1.85	(0.01)	1.97	(0.08)
	300	1.72	(0.09)*	1.82	(0.03)	2.02	(0.09)
	480	1.58	(0.05)**	1.84	(0.01)	2.05	(0.04)
	960	1.44	(0.10)**	1.81	(0.01)	1.99	(0.04)
130°C	min		min				
	0	2.07	(0.06)	1.84	(0.01)	2.09	(0.05)
	15	1.97	(0.06)	1.82	(0.02)	1.95	(0.11)
	30	1.87	(0.06)*	1.84	(0.01)	2.19	(0.12)
	45	2.02	(0.14)	1.81	(0.04)	1.91	(0.12)
	70	1.91	(0.06)*	1.83	(0.00)	2.11	(0.04)
	120	1.56	(0.40)	1.82	(0.02)	2.29	(0.05)

*: p<0.05 -paired Student's t test for yield difference between t0 time and t within each kinetic.

**: p<0.01—paired Student's t test for yield difference compared to t0 within each kinetic.

***: p<0.001—paired Student's t test for yield difference compared to t0 within each kinetic.

SD: standard deviation calculated with extracted DNA from 3 randomly-selected minicapsules.

**Table 2:** Quantity and quality of DNA extracted from encapsulated buffy coat assessed by spectrophotometric and fluorometric measurements.

Temperature	Storage time	Yield (μg)		A_260 nm_/ A_280 nm_		A_260 nm_/ A_230 nm_	
		Mean (SD)		Mean (SD)		Mean (SD)	
Ambient	months						
	0	1.45	(0.07)	1.84	(0.06)	1.44	(0.11)
	1.5	1.20	(0.08)	1.88	(0.01)	1.21	(0.20)
	2	1.17	(0.13)	1.84	(0.01)	1.18	(0.20)
	6	1.39	(0.03)	1.90	(0.01)	1.04	(0.08)
	12	1.12	(0.17)	1.83	(0.04)	1.05	(0.28)
	18	1.32	(ND)	1.87	(ND)	1.13	(ND)
50°C	months						
	0	28.90	(6.45)	1.87	(0.01)	2.02	(0.03)
	1	8.80	(7.85)*	1.87	(0.12)	1.23	(0.61)
	2	20.66	(3.69)	1.70	(0.26)	1.48	(0.13)
	3	35.26	(23.62)	1.86	(0.01)	1.71	(0.16)
	4	6.57	(1.26)**	1.82	(0.03)	1.30	(0.05)
	8	21.53	(7.42)	1.86	(0.01)	1.68	(0.09)
70°C	days						
	0	5.40	(0.81)	1.89	(0.15)	1.49	(0.16)
	7	7.47	(1.29)	1.95	(0.11)	1.48	(0.08)
	10	5.91	(0.91)	1.92	(0.08)	1.39	(0.08)
	17	7.01	(0.90)	1.95	(0.07)	1.50	(0.11)
	24	5.51	(0.54)	1.88	(0.07)	1.40	(0.06)
	30	6.26	(0.87)	1.92	(0.05)	1.42	(0.06)
110°C	min						
	0	4.31	(1.00)	1.87	(0.01)	2.04	(0.16)
	60	4.04	(0.31)	1.88	(0.01)	2.28	(0.55)
	180	4.21	(0.07)	1.88	(0.01)	1.92	(0.02)
	300	4.14	(0.31)	1.87	(0.01)	1.83	(0.09)
	480	4.07	(0.28)	1.87	(0.00)	1.84	(0.03)
	960	4.05	(0.50)	1.87	(0.02)	1.85	(0.20)
130°C	min						
	0	4.69	(0.67)	1.85	(0.02)	1.68	(0.15)
	15	3.63	(1.05)	1.83	(0.02)	1.67	(0.10)
	30	4.41	(0.29)	1.82	(0.01)	1.51	(0.01)
	45	4.55	(0.23)	1.82	(0.01)	1.60	(0.03)
	70	4.59	(0.06)	1.83	(0.01)	1.67	(0.03)
	120	4.44	(0.49)	1.80	(0.01)	1.61	(0.19)

*: p<0.05 -paired Student's t test for yield difference between t0 time and t within each kinetic.

**: p<0.01—paired Student's t test for yield difference compared to t0 within each kinetic.

SD: standard deviation calculated with extracted DNA from 3 randomly-selected minicapsules.

ND: non determined as measure corresponds to one minicapsule.

We recovered fair amounts of DNA from all the minicapsules, with a maximum of 10 μg from WBC minicapsules and of 35 μg from BC minicapsules. The high standard deviations for some time points of the 70°C and ambient temperature kinetics for WBC and for some time points of the 50°C for BC are probably due to variations linked to the extraction procedure (see Discussion). Such variations were also seen for the time 0 (t0) of the high temperature kinetics (here, we used the same batch of donor). Considering the whole set of kinetics at once, there are only a few time points for which the yield is significantly different from the time 0 yield. This suggests that the yield does not systematically decrease with the incubation time (see t-test in Tables 1 and 2, S1 Fig).

The A260 nm/ A280 nm ratios averaged 1.84 (±0.03) for WBC and 1.86 (±0.06) for BC (Tables 1 and 2) suggesting a low protein contamination. The A260 nm/ A230 nm ratios averaged 2.12 (±0.24) for WBC and 1.58 (±0.33) for BC (Tables 1 and 2). Although the average A260 nm/A280 nm ratios are not significantly different between WBC and BC extracted DNA, the A260 nm/ A230 nm ratio are (p<0.01), reflecting the invariable presence of contaminants in the extracted DNA samples extracted from BC lysates. This might be due to the initial presence of red blood cells or to a lack of extraction optimization for this source, or a combination thereof.

### Ambient temperature DNA stability in encapsulated buffy coat and white blood cells

**Fig 2:**
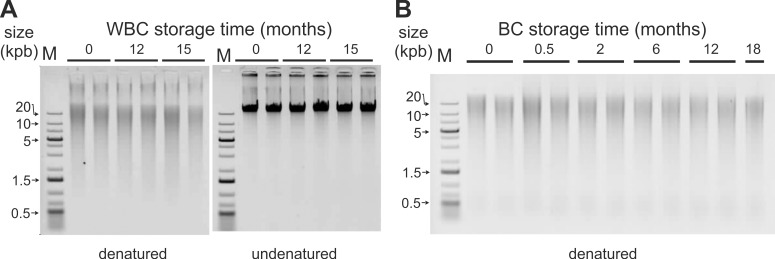
**Ambient temperature stability of DNA in encapsulated WBC (A) and BC (B).** Minicapsules of WBC and BC were stored for 15 months or 18 months, respectively, in the laboratory. At each time point, minicapsules were opened and samples rehydrated. Then, DNA was extracted and analyzed on non-denaturing AGE. DNA extracted from WBC were analyzed in two conditions: with or without heat-denaturation before AGE. DNA sizes are all > 20 kb. (A): extracted DNA from WBC after (left) or before (right) heat-denaturation, (B): extracted DNA from BC after heat-denaturation. M: double stranded DNA molecular weight marker.

We specifically set up analysis conditions to reveal DNA internal cuts through heat denaturation before electrophoresis analysis [37]. In some samples, according to pre-analytical conditions, heat-denaturation of extracted DNA samples at time 0 did reveal internal cuts in high molecular weight double strand DNA. However, storage of WBC minicapsules and BC minicapsules at ambient temperature did not exhibit additional cuts over 18 months for buffy coat samples (Fig 2B) and over 15 months for white blood cell samples (Fig 2A). DNA samples remained of high molecular weight over these storage periods.

### Effect of atmosphere exposure on encapsulated white blood cells and buffy coat DNA

**Fig 3:**
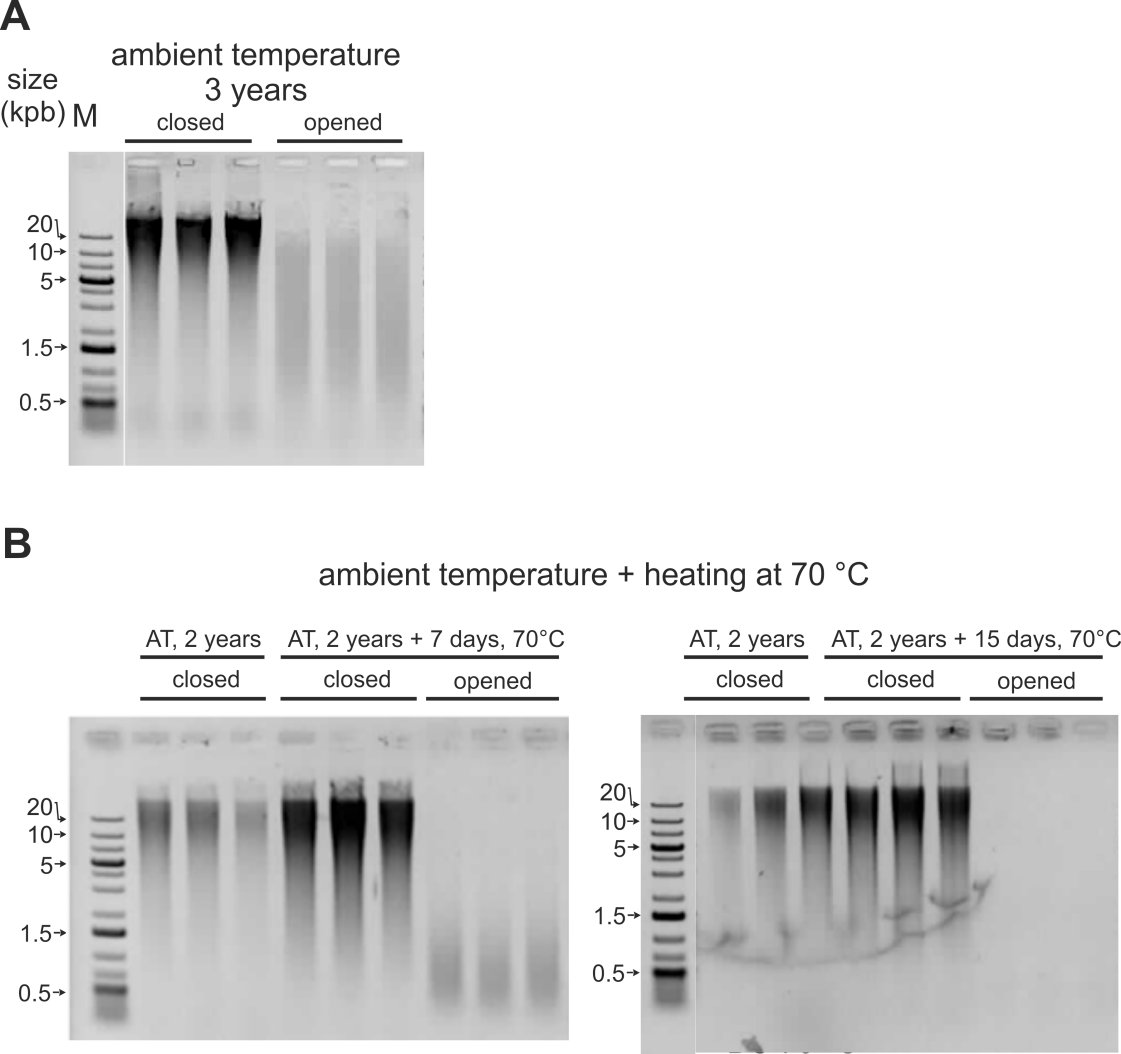
Adverse atmosphere effect on DNA stability of encapsulated white blood cells and buffy coat. (A) Opened and closed (standard Imagene conditions) BC minicapsules were left at ambient temperature for 36 months, (B) After a 24 months incubation at ambient temperature in Imagene standard condition, some minicapsules were opened. Both 2 years-old opened and closed WBC minicapsules were incubated for 7 and 15 days at 70°C under 50% relative humidity. Both WBC and BC samples (triplicates) were rehydrated, DNA extracted, heat-denatured to reveal internal cuts and analyzed on AGE.

We previously showed that after a few months storage at ambient temperature, degradation was apparent on purified DNA exposed to air [37]. In order to confirm this adverse effect on the DNA of stored WBC and BC lysates, samples in closed or opened minicapsules were incubated in presence or absence (standard Imagene conditions) of atmosphere exposure. Opened (exposed to atmosphere) and standard BC minicapsules were incubated for 36 months at ambient temperature. WBC minicapsules were incubated for 2 years at ambient temperature in Imagene standard conditions (closed). Then, some WBC minicapsules were opened and both closed and opened WBC minicapsules were heated at 70°C under air at 50% relative humidity. Fig 3A shows that DNA extracted from encapsulated BC remained of high molecular weight while it was degraded after a 36 month-exposure to air in ambient conditions: the DNA sizes decreased from > 20 kb to ~2kb. Fig 3B shows that DNA remained of high molecular weight after 24 months at RT (t0) in standard Imagene condition. Heating these 2 year-old WBC minicapsules for one or two weeks at 70°C under 50% relative humidity had no impact on DNA size in Imagene standard conditions, whereas DNA was degraded when exposed to 50% relative humidity.

### DNA degradation at elevated temperatures and Arrhenius plot

**Fig 4:**
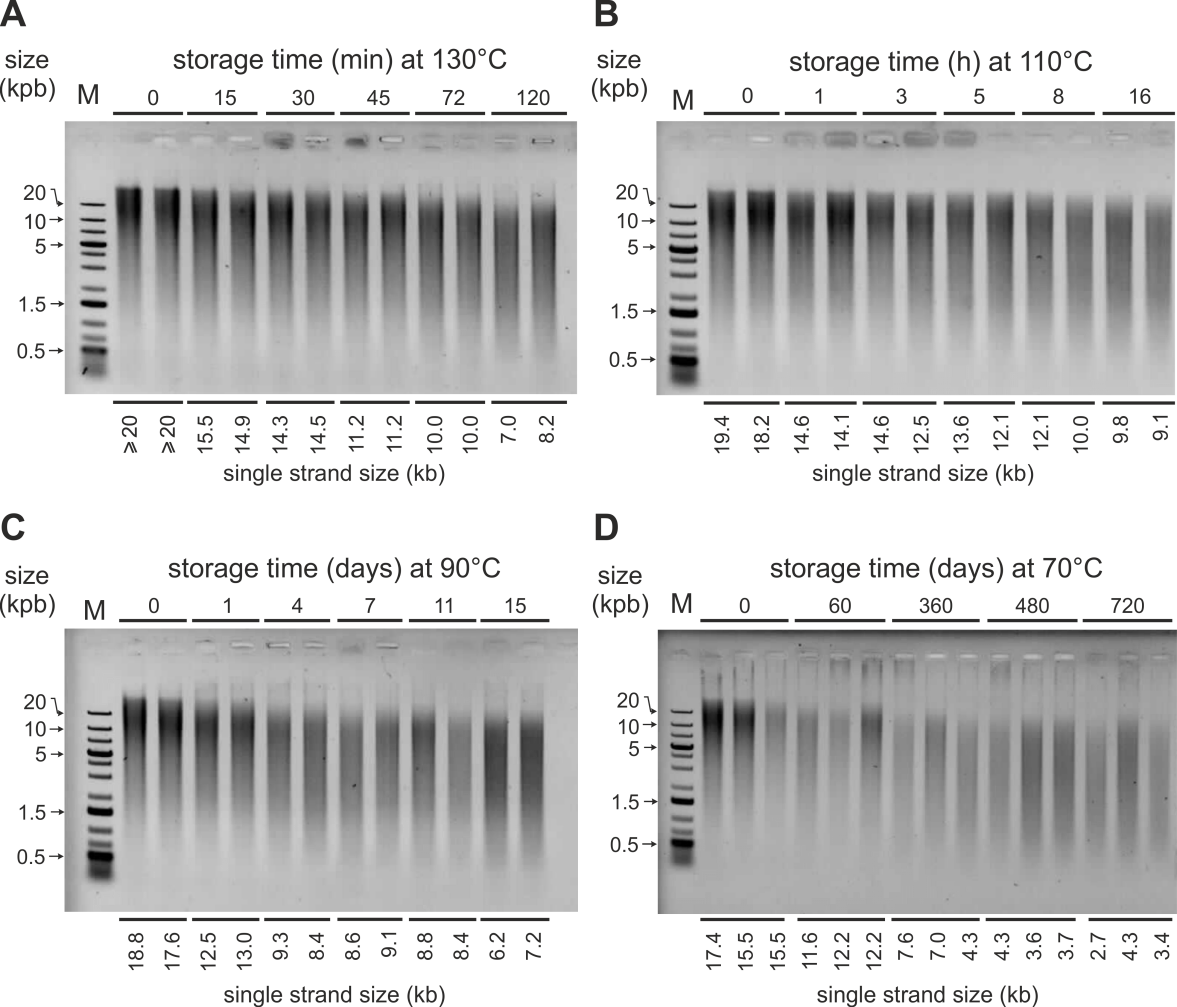
DNA degradation in heated encapsulated WBC. Representative electrophoresis gels: DNA samples extracted from white blood cells stored in Imagene minicapsules at the indicated temperatures (130°C, 110°C, 90°C, 70°C) and times were heat-denatured 5 min at 95°C to reveal internal cuts (single strand DNA size) and run on non-denaturating agarose gels. L_max_ values representing the average fragment size of DNA are indicated at the bottom of each lane. M: DNA molecular weight ladder; min: minute; h: hour.

**Fig 5:**
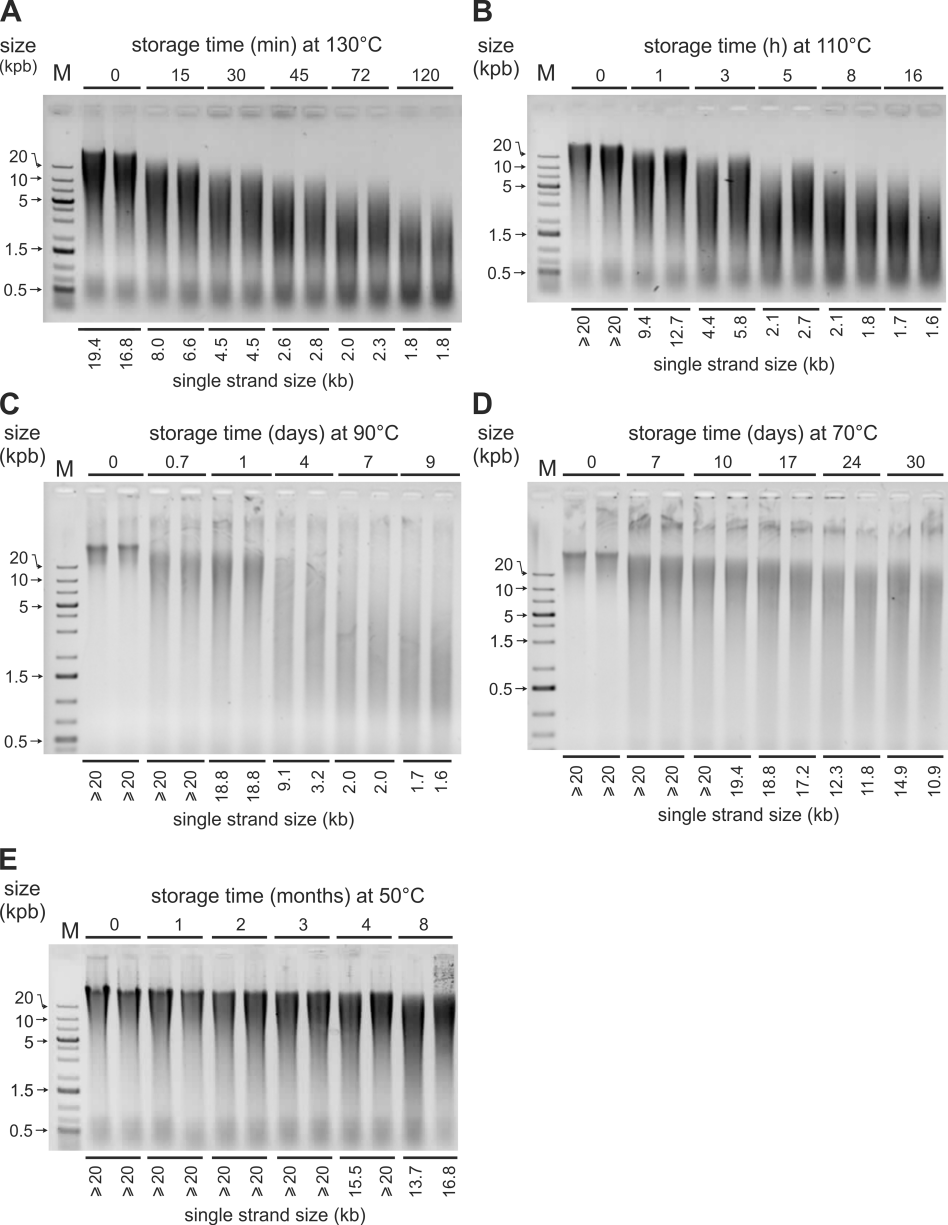
DNA degradation in heated encapsulated BC. Representative electrophoresis gels: DNA samples extracted from buffy coat stored in minicapsules at the indicated temperatures (130°C, 110°C, 90°C, 70°C) and times were heat-denatured 5 minutes at 95°C to reveal internal cuts (single strand DNA size) and run on non-denaturating agarose gels. L_max_ values representing average fragment size of DNA are indicated at the bottom of each lane. M: DNA molecular weight ladder; min: minute; h: hour.

From previous studies [10,14,38], DNA degradation in encapsulated WBC and BC was expected to be too slow at ambient temperature to be easily measurable. Therefore, in parallel, we set up accelerated aging kinetics at temperatures ranging from 50°C to 130°C. After extraction, the DNA samples were heat-denatured and run on non-denaturing agarose gels as seen on Fig 4 and Fig 5. The degradation rates were high enough, to enable us to measure L_max_ (see methods).

**Fig 6:**
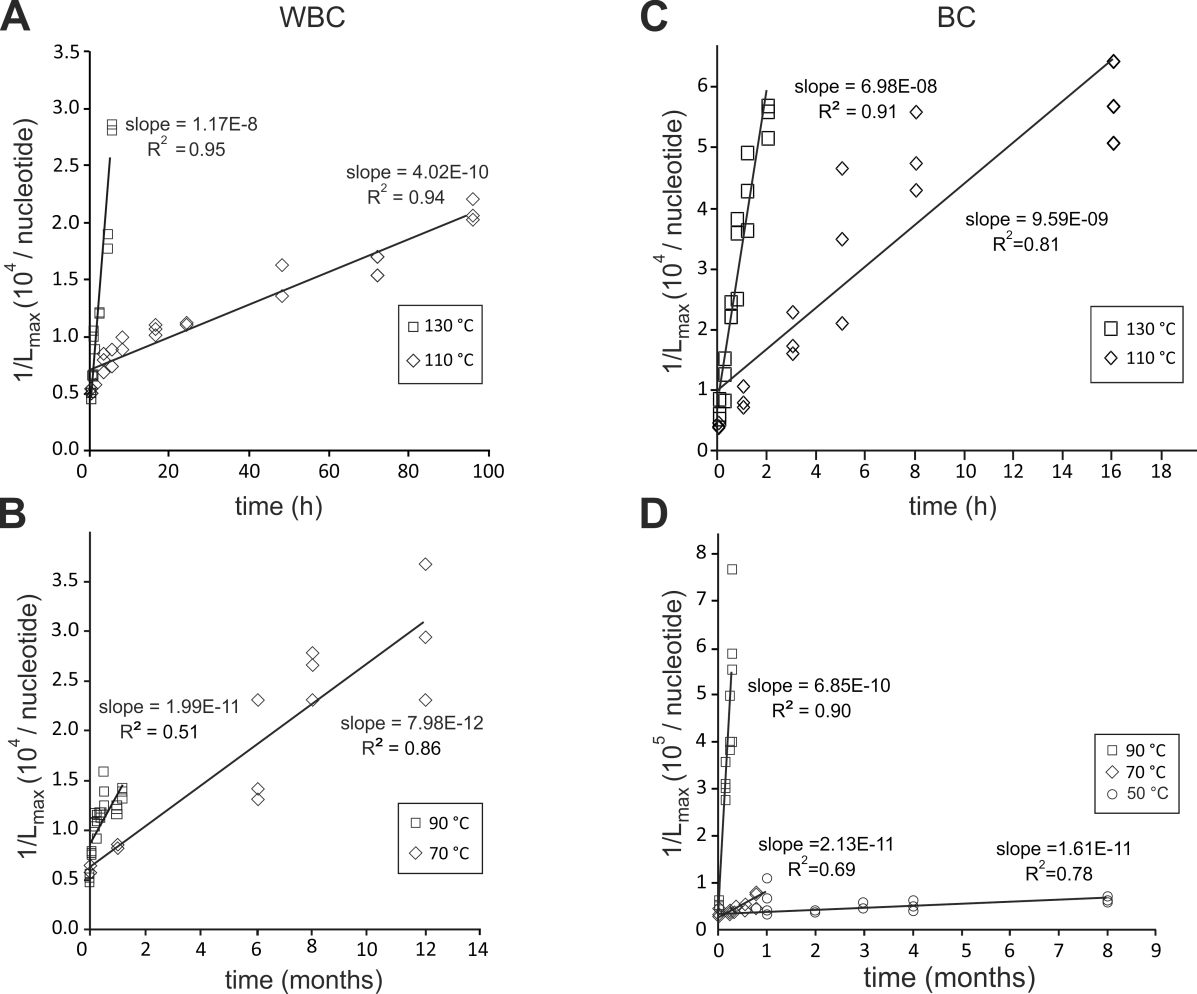
**Degradation kinetics of DNA extracted from heated WBC (A, B) and BC (C, D) minicapsules.** 1/L_max_ values were calculated for each time point of the kinetics from the L_max_ values measured on AGE (single size DNA strand). They were plotted as a function of the storage time at a given temperature: (A) DNA extracted from WBC minicapsules heated at 130°C and 110°C, (B) DNA extracted from WBC minicapsules heated at 90°C and 70°C, (C) DNA extracted from BC minicapsules heated at 130°C and 110°C, (D) DNA extracted from BC minicapsules heated at 50°C, 70°C and 90°C.

For measurable L_max_ (<20 kb), we plotted 1/L_max_ versus time at the different temperatures for the WBC and the BC lysates (Fig 6).

**Fig 7:**
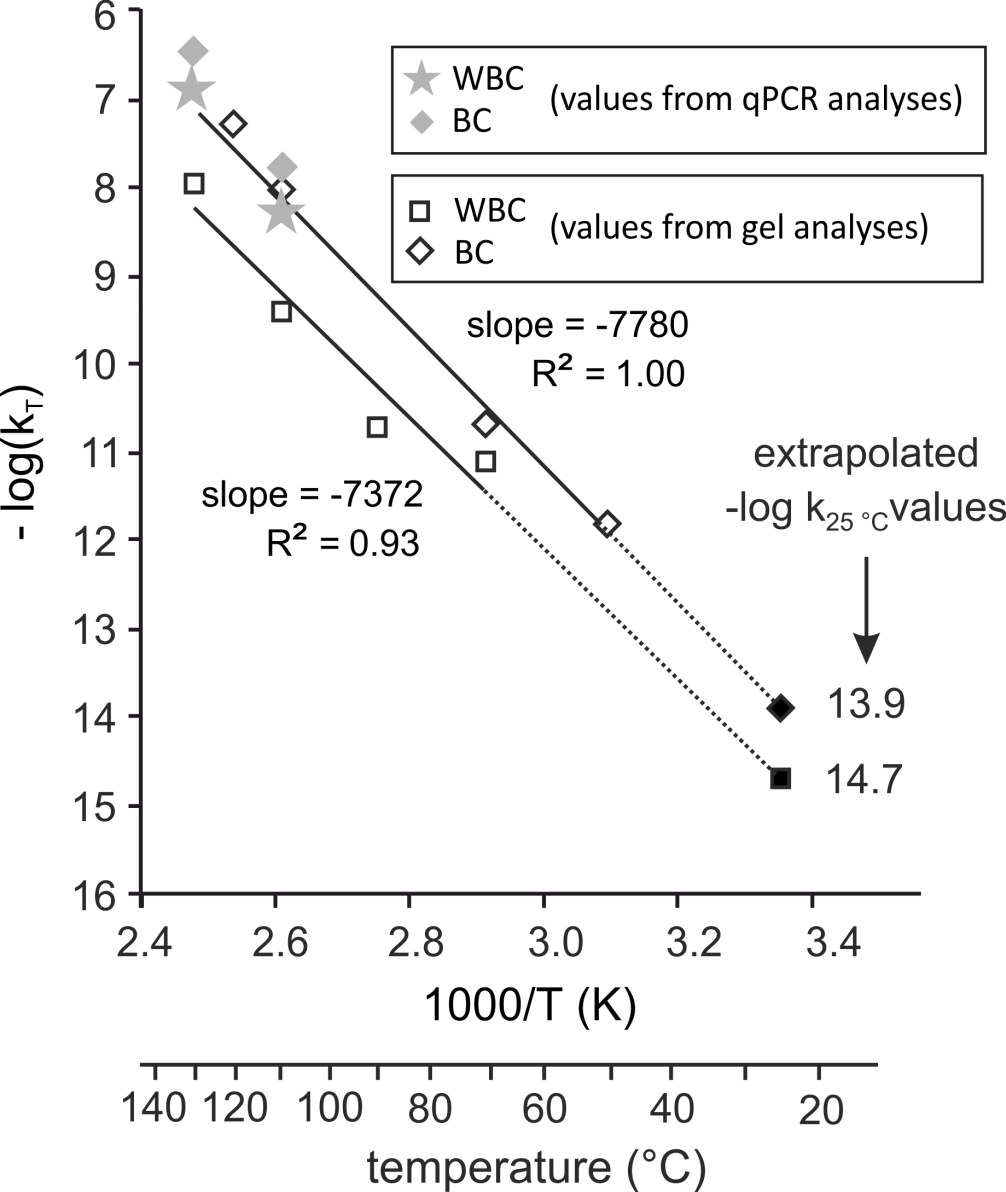
Arrhenius plots for DNA degradation of encapsulated WBC and BC. The DNA degradation rate constants, k_T_, were calculated either from qPCR analyses (grey symbols) or from AGE analyses (open symbols). The logarithms of k_T_, log(k_T_), were plotted as a function of the reverse of the absolute temperature (T). Correspondence between absolute temperature and temperature in°C are given on the lower x axis. The slope of–log(k_T_) as a function of 1/T is given for both WBC and BC minicapsules from gel analyses k_T_. Extrapolation values of log(k_25°C_) are given as “extrapolated–log k_25°C_ values” at 25°C from “gel analysis” k_T_. Open symbols: k_T_ values obtained from L_max_ measures on gel analyses. Grey symbols: k_T_ values obtained from qPCR analyses. Solid symbols: k_T_ extrapolated -log(k_25°C_) from gel analyses.

For each temperature, T, we determined the degradation rate constant, k_T_ from the slopes of the linear regressions according to Eq 1, indicated in Fig 6. We plotted -logk_T_ versus 1/T (K) (Arrhenius plot, Eq 2) (Fig 7).

The k_T_ data from gel electrophoresis could be fitted by the Arrhenius equation for both WBC and BC minicapsules, hence the DNA degradation rates at 25°C were evaluated by extrapolation. These extrapolated chain breaking rates (cuts per nucleotide per second) are 2.02x10^-15^ and 1.22x10^-14^ for WBC and BC, respectively. The corresponding apparent activation energies, calculated from the slopes of the Fig 7, are 138 kJ.mol^-1^ and 151 kJ.mol^-1^ for WBC and BC respectively.

### Amplification of DNA extracted from the stored blood-fractions

The experiments described above showed that the stored DNA breaking rate in minicapsules is extremely low. Although it is known that depurination and chain breaking is generally the fastest way of DNA degradation, we wanted to check that the encapsulated DNA does not hold modifications such as cross-links or oxidations that could prevent molecular analyses. Thus, we ran quantitative PCR targeting two different sized amplicons: 1064 bp and 93 bp in the *TAF1L* human gene.

**Fig 8:**
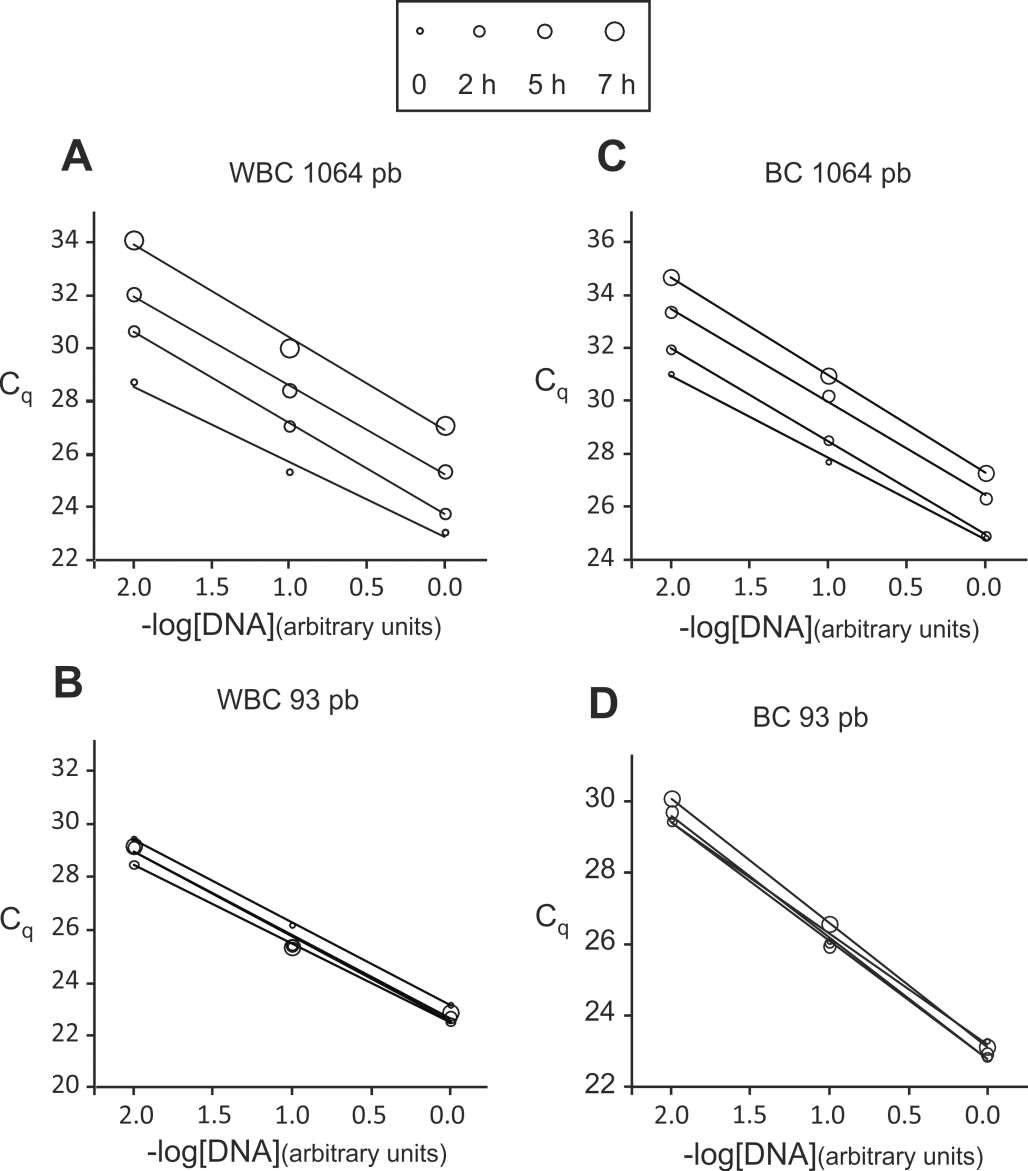
DNA amplification of extracted DNA from heated (130°C) encapsulated WBC and BC. The minicapsules were heated for 2 h, 5 h and 7 h at 130°C to strongly accelerate degradation. DNA was extracted from heated BC and WBC minicapsules and diluted for qPCR reactions. The targeted amplicon length was either 1064 pb (A, C) or 93 pb (B, D). Samples dilutions were as follows: non-diluted, 10-fold-diluted and 100-fold-diluted represented by 0.0, -1.0 and -2.0, respectively. (A, B) amplifications from WBC DNA, (C, D) amplifications from BC DNA.

We ran PCR amplifications on DNA extracted from WBC and BC heated minicapsules with three different concentrations generated by serial 10-fold dilutions to assess amplification efficiencies. A representative experiment is shown in Fig 8.

Fig 8 shows that DNA extracted from heated WBC and BC minicapsules could be amplified by PCR with no indication of the presence of inhibitors since the PCR efficiency was constant and near 100% for both amplicons regardless the time points and temperatures [39,40,41] (Table 3). Similar results were found for samples heated at 110°C.

**Table 3:** qPCR Amplification efficiencies for DNA extracted from heated WBC and BC minicapsules.

Heating temperatures (°C)	Amplification efficiencies of qPCR reactions with extracted DNA from WBC or BC minicapsules: mean (SD)							
	WBC				BC			
	amplicon size (bp)				amplicon size (bp)			
	1064		93		1064		93	
110	0.97	(0.05)	1.10	(0.05)	0.85	(0.04)	0.99	(0.04)
130	1.04	(0.15)	1.10	(0.05)	0.96	(0.10)	1.01	(0.07)

WBC: white blood cells.

BC: buffy coat.

bp: DNA base pair.

Mean (SD) = calculated by including the “t0”, “t1”, “t2”, “t3” time points for each temperature, each amplicon size and each blood product.

As expected, the Cq values for the 1064 pb amplicon (C1q) increased with degradation time while the Cq values for the 93 pb amplicon (C2q) exhibited little or no increase.

**Fig 9:**
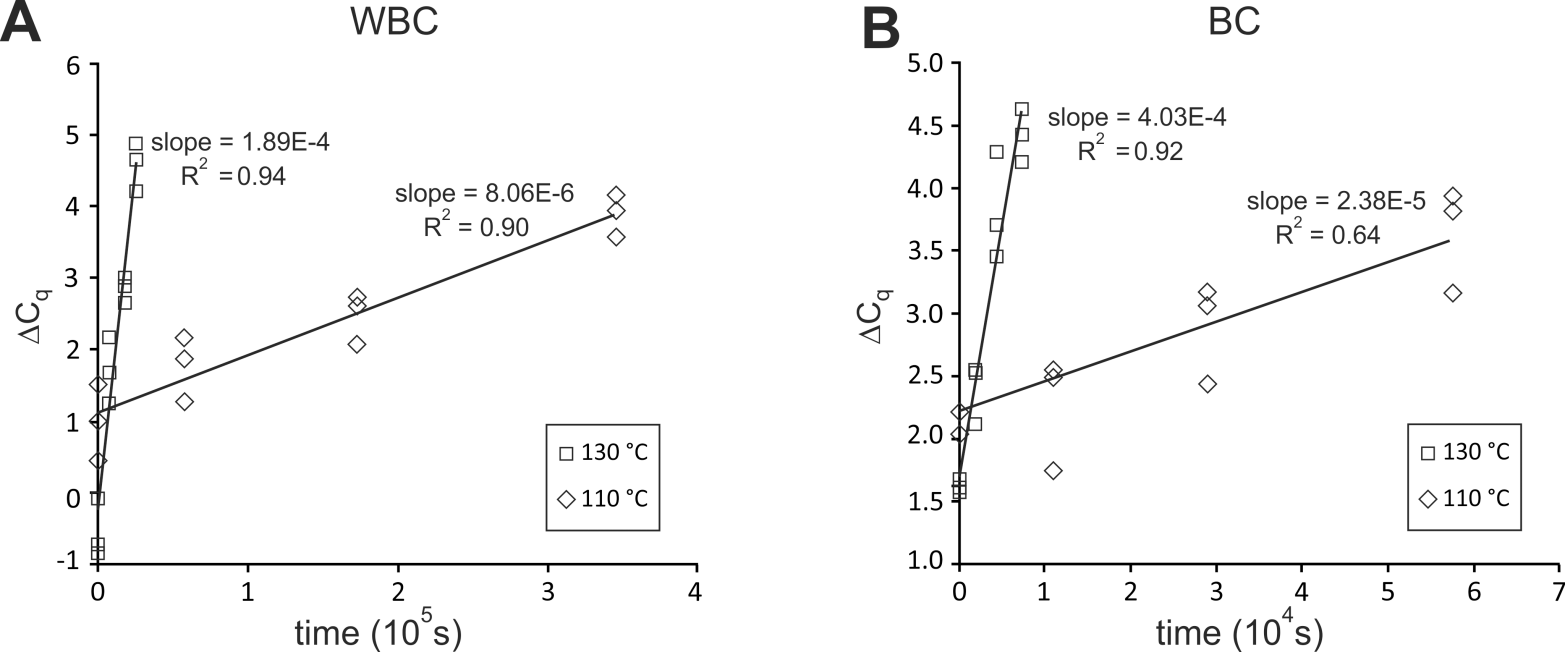
**Degradation kinetics of DNA extracted from heated WBC (A) and BC (B) minicapsules.** The ΔCq values were calculated by subtracting the Cq value of the “93 bp” amplicon from the Cq value of the “1064 bp” amplicon. This subtraction was done for each corresponding time point of the 130°C and 110°C kinetics and for both WBC and BC minicapsules. These values were plotted as a function of storage time at the indicated temperatures.

We used Eq (3) to estimate the degradation constant k_qPCR_ for 130°C and 110°C from the slopes of the straight lines obtained by plotting ΔCq values versus time (Fig 9).

The estimated k_qPCR_ values extracted from these data are presented in Table 4 and compared with k_gel_ (see discussion and Fig 7).

**Table 4:** qPCR Degradation rate constants, k_qPCR_, at 110°C and 130°C estimated with Eq (3) from qPCR data.

Heating temperatures (°C)	k_qPCR_ (number of cuts/nucleotide/second)	
	WBC	BC
110	5.75E-09	1.69E-08
130	1.34E-07	2.87E-07

## Discussion

The objective of this study was to assess the long term preservation at ambient temperature of DNA in lysates of blood-derived fractions protected by minicapsules in terms of recovery, integrity and usability.

### DNA extraction yields from WBC and BC minicapsules

The DNA quantities extracted from most of the lysates were in the expected range, around 3 μg, and depended mainly on the pool of donors. For example, the extraction yields were 8.63 μg (± 3.01) for the WBC minicapsules that went through ambient temperature kinetics while they were 1.93 (± 0.82) for the WBC minicapsules that went through high temperature kinetics because they come from a different pool of blood donors. In agreement with that, differences in DNA yields between WBC and BC were also observed.

For a given batch of minicapsules, we observed some variations but they were not correlated to either the temperature or the incubation time. So these differences were probably due to the expected differences in efficiencies of the lysis and extraction steps [42,43,44] rather than degradation or aggregation. Overall, these data indicate that there is no loss due to the minicapsule preparation or storage whatever the temperature. This can be opposed to the gradual decreases of extraction yields during frozen blood or buffy coat storage [45].

### DNA purity

Spectrophotometric analyses of extracted DNA showed consistent values close or equal to 1.8 for A260 nm/ A280 nm ratios indicating no detectable protein contamination regardless the storage temperature and duration.

A260 nm/ A230 nm ratios were also high for WBC and systematically lower for BC probably reflecting the fact that extractions are more challenging regarding contaminants when dealing with BC which were obviously contaminated by red blood cells [1,46,47,48][1,37,38,39].

### DNA amplifiability

As PCR is a technique universally used for DNA analysis or engineering, a series of qPCR reactions was performed to further validate the storage process. All extracted DNA samples behaved as expected in qPCR analyses with no indication of the presence of interfering residual contaminants (e.g. susceptible of affecting the polymerase enzyme performance).

### DNA stability in the WBC and BC minicapsules

As seen in Figs 2 and 3, at ambient temperature, no detectable DNA degradation occurred in the protected samples during the 3 year period of the study. Likewise, no aggregation was seen in these WBC and BC samples as evidenced by the absence of high molecular weight molecules in the gels or in the wells [25,49,50]. The recovery yields were as expected, further indicating that no important aggregation had occurred.

On the contrary, the samples left in the presence of air exhibited clear degradation and low yield as soon as after a 3-year storage (real time for WBC minicapsules and time equivalence for BC minicapsules). This could be due to DNA-DNA or DNA-protein crosslinks, aggregation or attacks by Maillard products as reported for stored dehydrated tissues or other biospecimens [51,52,53,54].

From the Arrhenius model (Fig 7), a 7 days-heating at 70°C corresponds to ~3 years at 25°C in minicapsules storage conditions. The effect of air was deleterious on the unprotected samples heated for 7 days and no DNA could even be extracted from samples heated after 15 days at 70°C. In addition, these WBC samples showed a brown color and were difficult to extract.

Accelerated degradation kinetics are commonly used to estimate the shelf life of molecules such as those used in the pharmaceutical industry. Therefore a series of degradation kinetics were run at temperatures ranging from 50°C to 130°C. The degradation rates corresponding to chain breaking events were calculated at each temperature and their dependence on temperature were found to follow Arrhenius' law for both WBC and BC in minicapsules. These rates correspond to ~6 cuts/10^6^ nucleotide/century and ~4 cuts/10^5^ nucleotide/century for DNA extracted from WBC and BC. Pure DNA k_25°C_ is about 1–40 cuts/10^5^ nucleotide/century in Bonnet *et al*. [10] conditions. From these figures, high molecular weight WBC and BC DNA would remain larger than 20 kb after at least one century at ambient temperature. In accordance with the model, no degradation could be seen in the samples stored at ambient temperature for this study.

We also calculated degradation rate constants k_qPCR_ values from qPCR data (Table 4). Differences between k_qPCR_ and k_gel_ are expected, first, because the AGE-determined k_T_ are apparent values and second, because the qPCR method takes more events than chain breakage into account. However, generally, this should not be significant since it is known that depurination and subsequent chain breaking is the main non enzymatic DNA degradation process.

In our study, k_qPCR_ and k_gel_ can be directly compared for BC as they come from the same batch of capsules processed in the same way. We found that calculated k_qPCR_ values for BC are of the same order of magnitude as that of the corresponding values determined from gel analyses (~2 and ~4 fold difference at 110°C and 130°C, respectively) and independently validate them.

For WBC, the differences are larger (8 and 23 fold for 110°C and 130°C, respectively). This can be explained by the fact that minicapsules for qPCR and AGE experiments were from different batches. This is not unexpected since in previous studies, we have experienced that parameters such as sample origin, encapsulation process and extraction protocols might induce some variability in the stability of stored DNAbut without impairing the general robustness of the encapsulated samples stability.

To the best of our knowledge, no Arrhenius model has been built and no degradation rate at ambient temperature have been estimated for alternative ambient temperature WBC or BC stabilizing product. In addition, the reported agarose gel analyses for competitor products are conducted in the absence of DNA denaturation so that they do not reveal internal cuts in the double stranded DNA. This strongly underestimates the real size of the DNA molecules and can even totally mask degradation as previously shown [37]. Therefore, no direct performance comparison can be made.

Finally, the activation energies for the degradation reactions, calculated from the Arrhenius plot are 141 kJ.mol^-1^ and 150 kJ.mol^-1^ for WBC and BC, respectively, in agreement to the 150–159 kJ.mol^-1^ and 153–156 kJ.mol^-1^ values which have been reported for the degradation of solid state plasmid DNA [10] and short single strand DNA [24], respectively.

Our determination of ambient temperature degradation rates relies on gel analyses which are blind to DNA alterations not involving chain breaks. Chain breaks come mainly from depurination. Those, especially in acidic conditions can accumulate in the DNA molecule, however, as we have previously shown, chain breaking occurs rapidly upon dehydration and do not accumulate in dehydrated DNA [10]. Oxidation of guanine is another event which does not lead to chain break, but in the absence of oxygen and especially water, oxidation is unlikely to occur [10]. Moreover, oxidation prevents the DNA chain elongation by DNA polymerase and so can be evidenced by PCR. As seen here, a major oxidation event can be excluded.

## Conclusions

As shown here, this innovative technology, through the combination of specific stabilization media and the permanent anhydrous and anoxic conditions, allows a reliable ambient temperature storage of white blood cells and buffy coat lysates as sources of genomic DNA.

Ambient temperature storage of blood-derived fractions, has many applications and impacts research and clinical practices:

- Biobanking strategy: importantly, when dealing with numerous samples, systematic DNA extraction might be too costly and postponing DNA extraction until needed requires to store the blood samples. Our procedure allowing a strong reduction in volumes appears as a valuable alternative to whole blood storage for DNA based analyses. Overall, it contributes to the sustainability of blood products collections from both operational and financial points of view.
- Biobanking sustainability: using minicapsules will improve storage efficiency by reducing storage costs and carbon dioxide production.
- External Quality Assessment (EQA) programs and Internal Quality Controls (IQC) programs of medical, clinical and diagnostic laboratories under ISO 15189: these programs often require large number of biological samples to be produced, stored possibly for years, sent worldwide and used on a routine basis as controls for standardization practices. The Imagene process allows the production of such large batches of identical materials whose analytical DNA properties will remain adequate for decades. This enables the comparison of method performances and analyses results with no additional cost linked to cold chain.

## Supporting information

S1 Fig Quantity and purity of DNA extracted from encapsulated WBC and BC. The quantity and purity of extracted DNA were assessed by UV spectrophotometric measurements. For each kinetic, we normalized the extraction yields of each time point to time 0. A Student’s t-test was performed for pair-wise comparison of DNA extraction yields throughout the heating kinetic and for pair-wise comparison of BC and WBC Absorbance means. *: p<0.05 -paired Student's t test for yield difference between t0 time and t within each kinetic. **: p<0.01—paired Student's t test for yield difference compared to t0 within each kinetic. ***: p<0.001—paired Student's t test for yield difference compared to t0 within each kinetic.
(TIF)
