# Supplementary figures and images for: High DNA stability in white blood cells and buffy coat lysates stored at ambient temperature under anoxic and anhydrous atmosphere
Source: PLoS One. 2017 Nov 30;12(11):e0188547. doi: 10.1371/journal.pone.0188547 (PMC5708797; doi:10.1371/journal.pone.0188547)

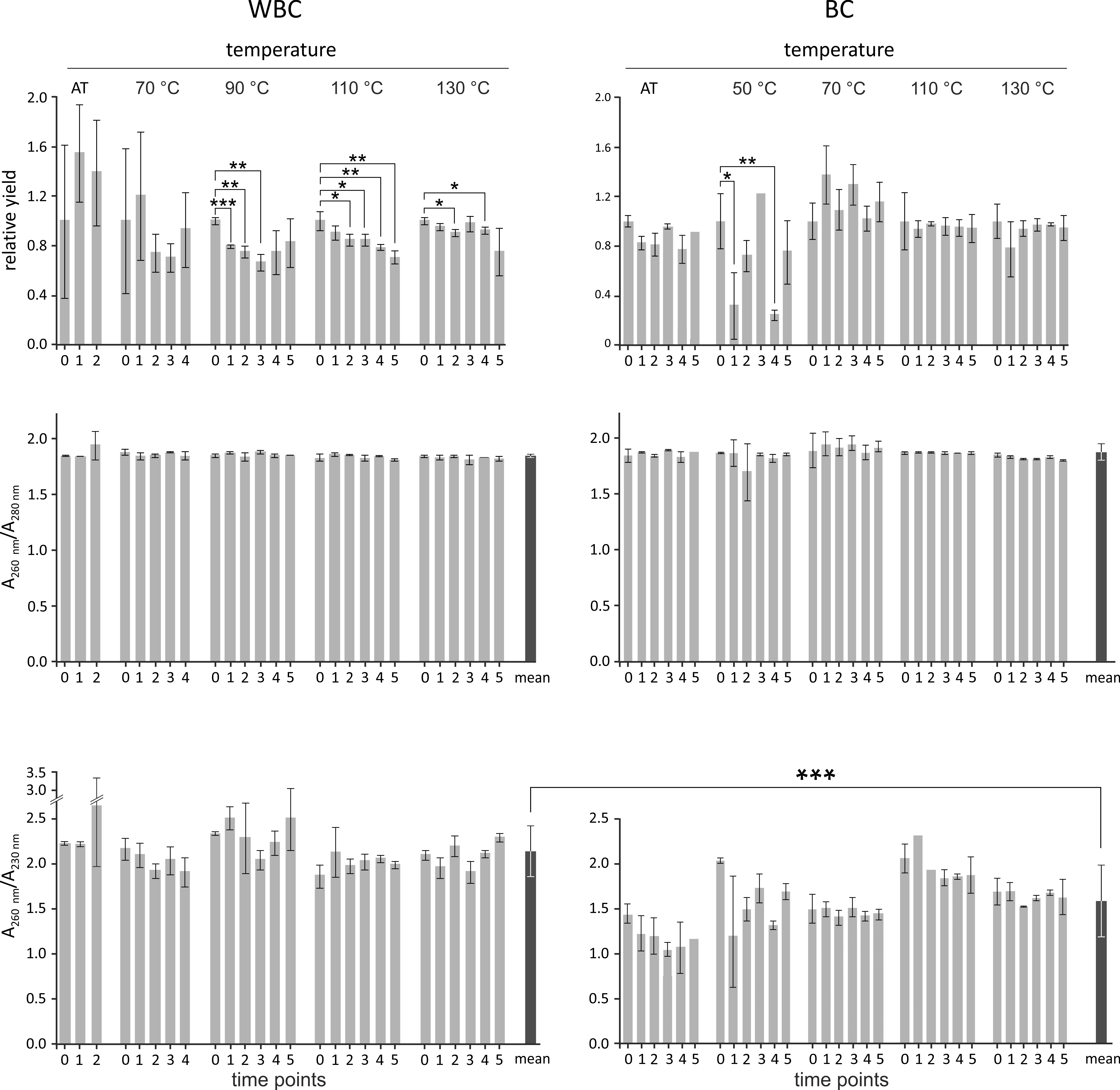

Supplement: S1 Fig — The quantity and purity of extracted DNA were assessed by UV spectrophotometric measurements. For each kinetic, we normalized the extraction yields of each time point to time 0. A Student’s t-test was performed for pair-wise comparison of DNA extraction yields throughout the heating kinetic and for pair-wise comparison of BC and WBC Absorbance means. *: p<0.05 -paired Student's t test for yield difference between t0 time and t within each kinetic. **: p<0.01—paired Student's t test for yield difference compared to t0 within each kinetic. ***: p<0.001—paired Student's t test for yield difference compared to t0 within each kinetic. (TIF) [file pone.0188547.s001.tif]
